# How to improve adherence to antidepressant treatments in patients with major depression: a psychoeducational consensus checklist

**DOI:** 10.1186/s12991-020-00306-2

**Published:** 2020-10-12

**Authors:** Bernardo Dell’Osso, Umberto Albert, Giuseppe Carrà, Maurizio Pompili, Maria Giulia Nanni, Massimo Pasquini, Nicola Poloni, Andrea Raballo, Fabio Sambataro, Gianluca Serafini, Caterina Viganò, Koen Demyttenaere, Roger S. McIntyre, Andrea Fiorillo

**Affiliations:** 1 Department of Biomedical and Clinical Sciences “Luigi Sacco”, University of Milan, Milan, Italy; 2 Department of Mental Health, ASST Fatebenefratelli-Sacco, Milan, Italy; 3 Aldo Ravelli’ Research Center for Neurotechnology and Experimental Brain Therapeutics, Department of Health Sciences, University of Milan Medical School, Milan, Italy; 4 Department of Psychiatry and Behavioral Sciences, Stanford University, Stanford, CA USA; 5 Department of Medicine, Surgery and Health Sciences, University of Trieste, Trieste, Italy; 6 Azienda Sanitaria Universitaria Giuliano Isontina–ASUGI, Clinica Psichiatrica, Trieste, Italy; 7 Department of Medicine and Surgery, University of Milan Bicocca, Milan, Italy; 8 Dept. of Neurosciences, Mental Health and Sensory Organs, Suicide Prevention Center, Sant‘Andrea Hospital, Sapienza University of Rome, Rome, Italy; 9 Institute of Psychiatry, Department of Biomedical and Specialty Surgical Sciences, University of Ferrara, Ferrara, Italy; 10 Department of Human Neurosciences-Faculty of Medicine and Dentistry-SAPIENZA University of Rome, Rome, Italy; 11 Department of Medicine and Surgery, Section of Psychiatry, University of Insubria, Varese, Italy; 12 Section of Psychiatry, Clinical Psychology and Rehabilitation, Department of Medicine, University of Perugia, Perugia, Italy; 13 Center for Translational, Phenomenological and Developmental Psychopathology, Perugia University Hospital, Perugia, Italy; 14 Section of Psychiatry, Department of Neuroscience, University of Padova, Padua, Italy; 15 Padua Neuroscience Center, University of Padova, Padua, Italy; 16 Institute of Psychiatry, Department of Biomedical and Specialty Surgical Sciences, University of Ferrara, Ferrara, Italy; 17 Department of Neuroscience, Rehabilitation, Ophthalmology, Genetics, Maternal and Child Health, Section of Psychiatry, University of Genoa, Genoa, Italy; 18 IRCCS Ospedale Policlinico San Martino, Genoa, Italy; 19 Center for Public Health Psychiatry, KU Leuven, Leuven, Belgium; 20 Campus Gasthuisberg, Universitair Psychiatrisch Centrum KU Leuven (UPC-KUL), Leuven, Belgium; 21 Mood Disorders Psychopharmacology Unit, University Health Network, Toronto, ON Canada; 22 Institute of Medical Science, University of Toronto, Toronto, ON Canada; 23 Department of Pharmacology, University of Toronto, Toronto, ON Canada; 24 Department of Psychiatry, University of Toronto, Toronto, ON Canada; 25 Brain and Cognition Discovery Foundation, Toronto, ON Canada; 26 Department of Psychiatry, University of Campania “L. Vanvitelli”, Naples, Italy

**Keywords:** Adherence, Antidepressants, Major depressive disorder, Consensus, Concordance

## Abstract

Studies conducted in primary care as well as in psychiatric settings show that more than half of patients suffering from major depressive disorder (MDD) have poor adherence to antidepressants. Patients prematurely discontinue antidepressant therapy for various reasons, including patient-related (e.g., misperceptions about antidepressants, side-effects, and lack of tolerability), clinician-related (e.g., insufficient instruction received by clinicians about the medication, lack of shared decision-making, and follow-up care), as well as structural factors (e.g., access, cost, and stigma). The high rate of poor adherence to antidepressant treatments provides the impetus for identifying factors that are contributing to noncompliance in an individual patient, to implement a careful education about this phenomenon. As adherence to antidepressants is one of the major unmet needs in MDD treatment, being associated with negative outcomes, we sought to identify a series of priorities to be discussed with persons with MDD with the larger aim to improve treatment adherence. To do so, we analyzed a series of epidemiological findings and clinical reasons for this phenomenon, and then proceeded to define through a multi-step consensus a set of recommendations to be provided by psychiatrists and other practitioners at the time of the first (prescription) visit with patients. Herein, we report the results of this initiative.

## Background

Major Depressive Disorder (MDD) is a prevalent, recurring and disabling condition that poses major challenges in the treatment of affected patients. Among critical issues related to MDD treatment, patients' poor adherence to antidepressant medications plays a crucial role in many cases of nonresponse, acute relapses, recurrences in the long term, and increased morbidity, comorbidity, and mortality [1]. It has been shown that depressive symptoms in MDD and mood disorders, in general, account for the majority of time spent ill despite availability of effective treatments [2]. In particular, analyses of long-time persistence of depressive symptoms in MDD patients show that earlier ages of onset is associated with greater symptom persistence, particularly in the youngest subjects [3]. Moreover, many studies showed that functional recovery takes longer than syndromal remission, mainly due to residual depressive symptoms [4], and quality-of-life deficits affect depressed subject for long periods of time [5], highlighting the need for adequate and persistent antidepressant treatment.

Adherence to medications has been described in two major components: persistence (i.e., taking the medication throughout the intended course of treatment) and compliance (with medical directions) [6]. Indeed, the term adherence puts more of a burden on the clinician to form a therapeutic alliance with the patient, to gain concordance with the patient on the therapeutic choice, which thereby increases behavioral compliance and, possibly, enhances the therapeutic effect of the administered medication [7]. Nevertheless, replicated evidence indicates that approximately half of patients receiving care in psychiatric and/or primary care settings are nonadherent to prescribed antidepressants [8]. For example, in a recent study, besides patients who did not complete the first 6 months of treatment continuation, over 50% of subjects who remained in treatment exhibited poor adherence [9]. It was also observed that approximately 25% of patients discontinue antidepressant treatment within 1 month of treatment, and within 3 months of initiating therapy in the 44% of cases [10].

Nonadherence to antidepressants is a multifactorial phenomenon including both patient-related (e.g., concerns about side-effects, costs of medications, fear of addiction, and cultural and attitudinal issues) and clinician-related factors (e.g., lack of adequate patient education and shared decision-making, and poor follow-up) [8, 11, 12]. Therefore, strategies to promote adherence should address issues in prescribers’ attitudes and training [7, 11, 13], as patients' nonadherence to antidepressant medications may also reflect physicians' quality of care [14].

In fact, physician-specific issues including poor patient education, lack of shared decision-making, prescription of inadequate dosages of antidepressants, and lack of follow-up care are all aspects that physicians need to control to improve patient’s adherence, since they represent some of the main obstacles to adequate antidepressant treatments [10]. Due to evidence showing that the modality in which antidepressants are initially prescribed concurs to predict patient’s treatment adherence and outcome [15], the first antidepressant prescription represents the most important occasion to provide patients with adequate information on medications, side-effects, expectations, therapy duration, and follow-up. To this regard, it has been recently pointed out that only a minority of patients, who discontinue antidepressant treatment after the first prescription, subsequently complete an adequate treatment course within the following year [16]. Therefore, initiatives to promote adherence to appropriate antidepressant treatment should be taken *in primis* during their first prescription [16].

Based on the above, as in Italy reported antidepressant adherence rates are unsatisfactory and consistent with the aforementioned studies [17], we aimed to establish a consensus on the essential points clinicians which should discuss with MDD patients when antidepressant medications are first prescribed. We therefore used the results of this consensus to inform a psychoeducation module to be used by practicing clinicians at point of care when prescribing antidepressants, herein presented and discussed.

## Methods

A group of academic psychiatrists met on three different occasions, in October 2018, March 2019, and at the end of 2019 with the intent to discuss antidepressant adherence issues related to epidemiologic and causal aspects, and to elaborate successful strategies for promoting treatment adherence in patients suffering from MDD during their first antidepressant prescription visit.

The working groups included a focus group of academic psychiatrists (*n* = 7), a steering committee of academic psychiatrists (*n* = 5), a larger discussion panel of academic psychiatrists (*n* = 21), and an International panel of expert psychiatrists (*n* = 2, one from Canada and one from Europe). In particular, the first meeting was structured in two phases: during the initial part, the focus group presented and discussed the results of a systematic review within the following databases (Cochrane Library, PubMed, and Medline) performed up to October 2018 for relevant articles published in the area of antidepressants adherence through the terms “adherence”, “compliance”, “antidepressant”, “treatment”, “therapy”, “tolerability”, “outcomes”, “persistence”, “depression”, “MDD”, “patient”, “physician”, “psychiatrist”, and “general practitioner”. At the end of the discussion, the focus group identified a series of prioritary issues in the field, and then proceeded to elaborate a preliminary consensus checklist. During the second part of the first meeting, the preliminary checklist was presented and discussed at the presence of the whole working group (focus group, steering committee, discussion panel and international panel) for validation of scientific content, integration, and approval. The revised contents of the checklist were further discussed and approved during the second meeting (March 2019). The steering committee gathered for the third meeting at the end of 2019 with the aim of reviewing and confirming the definitive version of the checklist. Finally, the checklist was circulated to the whole working group for preliminary testing of feasibility and usability in clinical practice.

## Results

**Fig. 1:**
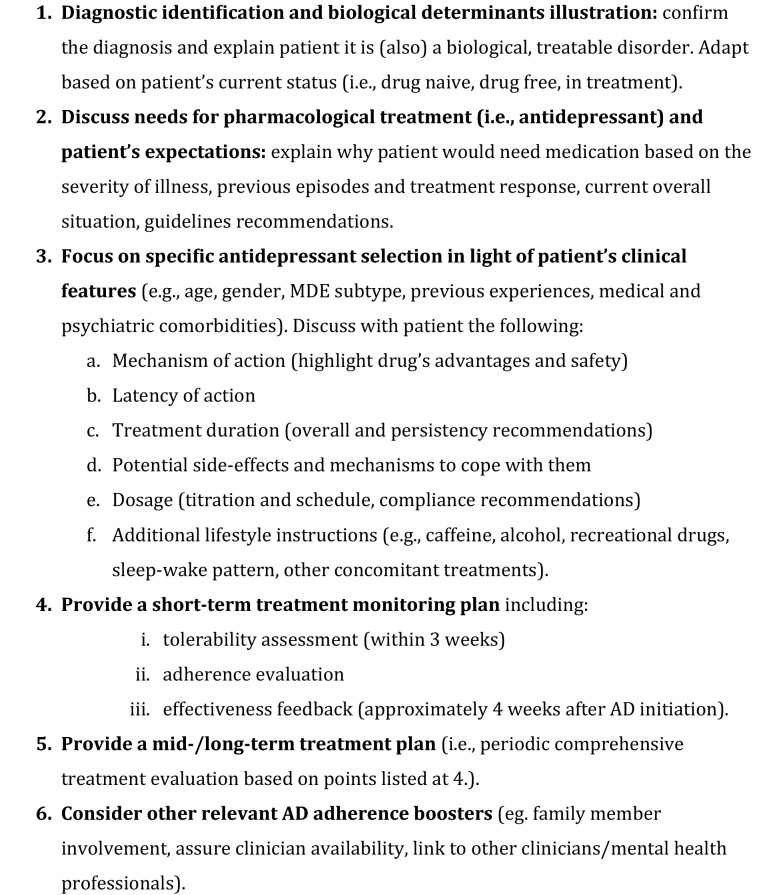
Pro-Antidepressant Adherence Checklist (PAAC). Checklist of items to be presented at the final part of the first visit with patients with major depressive disorder (MDD) and current major depressive episode (MDE) who might benefit from AD treatment (duration: 20–30 min)

Based on available literature and their clinical experience, consensus participants identified a series of sequential priorities to be addressed by prescribing clinicians when they first prescribe antidepressant medication to their MDD patients. These issues are summarized and included in Fig. 1 in the form of checklist (pro-antidepressant adherence checklist) and herein presented. The final level of consensus was unanimous among participants for all the issues presented.

### Provide a diagnostic framework and illustrate illness’ biological determinants

Consensus participants agreed over the need to provide patients with a clear establishment of diagnosis and related symptoms, based on currently available nosological systems [18, 19], as an essential step to highlight the indication and the usefulness of antidepressants, according to treatment guidelines [20]. In particular, consensus participants deemed necessary informing patients about the biological determinants of MDD [21], linking these to the antidepressants’ mechanism of action, to clarify why and how these medications can mitigate MDD symptoms. Participants agreed that this issue needs to be adapted to patients’ cultural/educational background and their cognitive and emotional status, aiming to find the right balance between being uselessly detailed and excessively superficial. In addition, the way clinicians may approach this issue depends on patient’s previous experience, if any, with antidepressants (i.e., drug-naive or drug-free condition or currently under another treatment) as well as to specific conditions (e.g., pregnancy, menopause, and geriatric depression).

### Discuss the need for pharmacological treatment (i.e., antidepressant) and patient’s expectations

Participants agreed on the importance of discussing the need for pharmacological treatment with patients, represented by antidepressant medications, in the context of the current major depressive episode (MDE), based on a series of different aspects. These are represented by—but not limited to—patient’s illness severity, history of previous episodes, suicide attempts and treatment response, current overall situation in terms of functional impairment, and guidelines recommendations. While addressing these points, it is important to have patients disclose their expectations and goals regarding treatment efficacy, tolerability, dosage, latency of action, and overall duration [22, 23].

### Select a specific antidepressant in light of patient’s clinical characteristics

Consensus participants convened that antidepressant selection represents the key issue in relation to treatment adherence and outcome. In fact, after explaining why the clinician has selected a particular antidepressant based on patient’s characteristics, including among others: age, gender, MDE subtype and specific status (pregnancy, postpartum, geriatric, adolescent, etc.), previous experience with other/same antidepressant, and medical and psychiatric comorbidities, it is important to provide essential information on the specific prescribed medication. This should include the following aspects:

- Mechanism of action (highlight drug’s pros and cons over other compounds patient may have already taken and safety issues);
- Latency of action (to provide patients with realistic expectations);
- Treatment duration (overall and persistency recommendations);
- Potential side-effects and mechanisms to cope with them;
- Dosage (titration and schedule, and compliance recommendations);
- Additional lifestyle instructions (e.g., moderate caffeine and alcohol intake, avoid use of recreational drugs, develop sleep hygiene, and integration with other concomitant treatments).

Consensus participants highlighted the importance of discussing the above-mentioned points in a shared decision-making perspective, as this approach can profoundly impact antidepressant adherence [24].

### Provide a short-term treatment monitoring plan

After discussing antidepressant choice and its characteristics, it is important to provide an initial short-term treatment plan, indicating that patients are expected to be visited within the subsequent 3 weeks. The first follow-up visit is intended to mainly assess patient’s tolerability and treatment adherence and, possibly, initial effectiveness, even though 4 weeks, depending on titration schedule, may be needed to detect first signals of response. Patients are supposed to appreciate receiving information about how to contact their clinician in case of necessity between the first prescription visit and the first follow-up visit.

### Provide a mid-/long-term treatment plan

Should excessive emphasis on treatment-related issues beyond the short term (in the context of the first visit) represent an overburden in relation to the patient’s status, it is very important to mention the need to plan periodic comprehensive treatment evaluations through subsequent visits. In fact, this issue could be of particular relevance to prevent patients from autonomously modifying the antidepressant dosage or prematurely interrupting treatment as they start feeling better, therefore exposing themselves to the risk of a relapse. The post-acute follow-up visits will explore antidepressant effectiveness and tolerability, as well as patient’s response, functional improvement, and overall expectation.

### Consider other relevant antidepressant adherence boosters

A series of other issues, not directly related to the specific antidepressant compound, can indeed represent important elements in boosting the overall treatment adherence. Clinicians may want to briefly discuss these in the final part of their first prescription visit. Among others, factors that can reinforce treatment adherence in the short-to-long term include: the possibility of involving caregivers/family members within the treatment plan, establishing links with other key health professionals previously involved (e.g., psychologist, family physician) [25], and assuring clinician’s availability in case of necessity (e.g., work phone, mail).

From preliminary investigational testing in their clinical practice, consensus participants acknowledged the discussion of the items included in the checklist as more suitable at the end of the visit, when an initial connection with the patient has already been established, and that the implementation of the checklist accounts for an overall duration of 20–30 min. Furthermore, the checklist was found to be useful and well received by both patients and their relatives, who also have acknowledged its importance in the therapeutic alliance process.

## Discussion

The present psychoeducational checklist represents the outcome of multiple meetings where participants, according to their clinical experience and based on available literature, had elaborated and discussed various issues deemed essential in relation to antidepressant adherence, with the aim of defining a series of priorities to be discussed by clinicians with their patients during the first prescription visit, and therefore promoting subsequent antidepressant adherence.

As previously stressed, poor adherence to antidepressants in patients with MDD has been consistently reported over at least the last 20 years, in different contexts and settings [26, 27].

Indeed, adherence to antidepressants in MDD patients, and more likely, adherence to psychotropics in patients suffering from psychiatric disorders, is a multifaceted issue and depends on multiple factors that have been clustered mainly into patient-related and clinician-related ones (among others) for convenience [8, 11, 12]. While such a distinction is still useful for research purposes, a mutual and reciprocal relationship between the above-mentioned factors and the quality of communication between clinician and patient is more likely to occur in clinical practice.

In addition, the quality and accessibility of information provided during the first prescription visit can make the difference in improving treatment adherence [28].

While every clinician has his or her own approach in dealing with patients suffering from MDD, and providing them with essential information regarding their prescribed medication (particularly antidepressants), we believe that following some basic, sequential steps can help clinicians remember specific aspects of particular importance in relation to adherence. While this approach can certainly be of benefit for psychiatrists, especially for those in training (including residents) and in early career, it can also be very useful for other clinicians who frequently prescribe antidepressants, including general practitioners, neurologists, geriatrists, and clinical psychologists [29, 30].

As a result of the consensus, on the basis of their clinical experience and available literature, participants identified six major issues to be discussed with MDD patients over the course of their first antidepressant prescription visit.

Consensus participants strongly believed that adequately informing patients about the MDD diagnosis and its biological determinants (and neurochemical correlates) represent the first key issue for promoting antidepressant adherence and achieving sustained remission [19]. Indeed, such a process needs to be developed while assessing patients’ individual perceptions about depression and its treatment, as these factors were found to strongly influence adherence, at initiation, implementation, and discontinuation phases of adherence to antidepressants [31].

The second issue to be discussed with patients to promote adherence to antidepressants was the need of pharmacological treatment in relation to their specific condition. Consensus participants, in fact, believed that after having formulated an MDD diagnosis and mentioned its biological underpinnings, clinicians need to illustrate the reasons why antidepressants are necessary in relation to patient’ status. This second issue aims at approaching patients’ specific condition, represented by their current MDE; therefore, information provided at this point by the clinician should vary according to specific characteristics. Severity of illness represents one of the most important factors. For instance, it has been shown that for patients with very severe depression, the benefit of antidepressants over placebo is substantial [32]. Other important aspects that should be discussed at this time of the visit include the presence of previous MDEs, as recurrent depression is a long-term condition that needs to be treated pharmacologically to reduce the risk of new episodes [33, 34]. Other relevant elements to be considered in relation to the recommended use of antidepressants include the presence of specific types of depression (e.g., those occurring in the elderly or pregnancy), as well as the patient’s functional impairment. As previously mentioned, this information needs to be provided while addressing patient’s expectations, beliefs, and previous experiences.

The third point addresses specific antidepressant choice based on the patient’s characteristics and represents the most articulated section for clinicians, because they are required to provide information regarding the drug’s mechanism of action, latency of action, treatment duration, potential side-effects, dosage, and additional lifestyle instructions. While discussing all these items, tolerability and side-effects certainly represent key issues to attenuate and possibly avoid poor adherence to antidepressants [35, 36]. Patients with poor adherence due to side-effects, in fact, are likely to show poor treatment response, and to drop out before having obtained any benefit [37]. Therefore, consensus participants strongly believed that clinicians need to pay particular attention in discussing the most frequent side-effects of a given antidepressant and the best ways to cope with them [38].

The fourth and fifth points intend to emphasize the longitudinal perspective of antidepressant treatments. As mentioned before, it is important to discuss with patients the importance of maintaining antidepressant treatment beyond the short term, given the risk of relapse and recurrences of MDD [39]. In addition, the discussion of these issues is aimed at engaging and committing patients to their prescribing clinicians to make informed decisions about antidepressants, particularly in case of interruption. In this perspective, in fact, it has been reported how patients tend to become expert at managing their depressive condition and the use of antidepressants through a process of trial-and-error, which typically follows a period of experimentation where it is not uncommon for the patient to stop and restart medications, often several times [40].

Finally, in the final point, consensus participants considered a series of other issues, not directly related to the antidepressant choice, though still capable of boosting the overall treatment adherence. Namely, the possibility of involving caregivers/significant family members [41] within the treatment plan, establishing a link with other previously involved key health professionals (psychologist, family physician) [25], and assuring clinician availability in case of necessity (work phone, mail) are all factors that can reinforce treatment adherence in the short- to long-term [42].

Consensus participants agreed on approaching the items included in the checklist not as separate—but rather as interconnected domains—with some degree of overlap.

While some clinicians may believe that the approach proposed in the checklist may be too over-inclusive and excessively time consuming, they should remember that often patients experience frustration with their health care providers, including feeling rushed and perceiving visit quality lacking, which can negatively impact antidepressant adherence, as reported by a recent International survey [43].

## Conclusions

While the present checklist is still under validation in a representative sample of MDD patients, and its putative usefulness is being tested in clinical practice, its preliminary and investigational use among consensus participants was found to be accessible and well received. Furthermore, since the checklist might help patients obtaining a more favorable disease experience, in a personalized medicine perspective, consensus participants speculated the checklist might be particularly useful for patients undergoing their first MDE or, more generally, for those who never went through medical treatment before.

In terms of methodological limitations, the items included in the checklist were generated by a consensus of academic psychiatrists who, despite being involved in the clinical practice of MDD patients in terms of diagnosis and treatment, may not necessarily meet the expectations and share the same points of view of other nonacademic psychiatrists and clinicians. Ultimately, results on validation of the checklist are needed to ultimately support its use in clinical practice. Nonetheless, the checklist reflects rather common-sensual aspects that are clearly relevant in psychoeducational terms and echoes typical explanatory procedures already broadly adopted in somatic medicine.

Since poor antidepressant treatment adherence represents a contributing factor in inadequate MDD treatment, with a corollary risk of chronicity and refractory and life-threatening outcomes, clinicians—particularly antidepressant prescribers—should focus their efforts in handling this condition with the same care as an initial malignant lesion [44] and, therefore, pay a special attention to treatment adherence to maximize the effectiveness of antidepressant treatment.

## Data Availability

Data sharing is not applicable to this article as no dataset was generated or analyzed during the current study.
